# Rapid prototyping of PMMA-based microfluidic spheroid-on-a-chip models using micromilling and vapour-assisted thermal bonding

**DOI:** 10.1038/s41598-024-53266-y

**Published:** 2024-02-03

**Authors:** Monieb A. M. Ahmed, Klaudia M. Jurczak, N. Scott Lynn, Jean-Paul S. H. Mulder, Elisabeth M. J. Verpoorte, Anika Nagelkerke

**Affiliations:** 1 Pharmaceutical Analysis, Groningen Research Institute of Pharmacy, University of Groningen, Groningen, The Netherlands; 2 W.J. Kolff Institute for Biomedical Engineering and Materials Science, University Medical Center Groningen, Groningen, The Netherlands; 3 Department of Surgery, University Medical Center Groningen, Groningen, The Netherlands; 4 Institute of Physics, Czech Academy of Sciences, Prague, Czech Republic

**Keywords:** Biomedical engineering, Cancer models

## Abstract

The application of microfluidic devices as next-generation cell and tissue culture systems has increased impressively in the last decades. With that, a plethora of materials as well as fabrication methods for these devices have emerged. Here, we describe the rapid prototyping of microfluidic devices, using micromilling and vapour-assisted thermal bonding of polymethyl methacrylate (PMMA), to create a spheroid-on-a-chip culture system. Surface roughness of the micromilled structures was assessed using scanning electron microscopy (SEM) and atomic force microscopy (AFM), showing that the fabrication procedure can impact the surface quality of micromilled substrates with milling tracks that can be readily observed in micromilled channels. A roughness of approximately 153 nm was created. Chloroform vapour-assisted bonding was used for simultaneous surface smoothing and bonding. A 30-s treatment with chloroform-vapour was able to reduce the surface roughness and smooth it to approximately 39 nm roughness. Subsequent bonding of multilayer PMMA-based microfluidic chips created a durable assembly, as shown by tensile testing. MDA-MB-231 breast cancer cells were cultured as multicellular tumour spheroids in the device and their characteristics evaluated using immunofluorescence staining. Spheroids could be successfully maintained for at least three weeks. They consisted of a characteristic hypoxic core, along with expression of the quiescence marker, p27^kip1^. This core was surrounded by a ring of Ki67-positive, proliferative cells. Overall, the method described represents a versatile approach to generate microfluidic devices compatible with biological applications.

## Introduction

In recent years, the use of microfluidic model systems has expanded in biological research owing in part to their versatile and dynamic nature, as well as innovations in microengineering techniques^1,2^. The continuous advancements in microfluidic systems, resulting from significant improvements in microfabrication techniques and material selection, have led to increased complexity and functionality of these systems. For instance, the introduction of polydimethylsiloxane (PDMS) for manufacturing microfluidic chips was primarily the result of the development of soft lithography and replica moulding fabrication methods^3–5^. Soft lithography has provided an attainable and attractive alternative to fabricate microfluidic devices, overcoming the iterative photolithography process of Silicon (Si) or the time-consuming etching of glass in earlier microfluidic designs^6,7^. As such, it has become increasingly evident that appropriate material selection, along with suitable fabrication methods, are integral to the evolution of microfluidic models and the emulation of biological structures.

Despite its advantages, the use of PDMS is associated with inherent challenges that undermine the accuracy of PDMS-based microfluidic devices particularly when studying molecular transport, drug uptake or toxicity^8–10^. Thermoplastic polymers have emerged as a viable option for developing microfluidic models that can overcome the limitations of PDMS^11,12^. Specifically, materials like polymethyl methacrylate (PMMA), cyclic olefin copolymer (COC), polycarbonate (PC), and polystyrene (PS) are highly desirable due to their ability to eliminate autofluorescence, leaching, and molecular absorption^13^. To date, thermoplastics have been utilised for designing different microfluidic chip systems spanning a wide range of applications^14^. For example, PMMA-based microfluidic chips have been used in drug testing, for studying tumour cell metastasis and for DNA separation^15–17^. Other thermoplastics including COC, PC and PS have been also used in manufacturing microfluidic devices^18,19^.

Thermoplastic polymers can be manufactured using a number of strategies, one of which is micromilling. Micromilling provides a multifaceted, low cost and rapid design-to-prototype microfabrication method^20,21^. Computer numerical control (CNC) micromilling machines can create different geometrical structures in thermoplastics within minutes using miniature cutting tools, such as endmills or milling tools, providing an ideal prototyping platform for microfluidic models^21^. Micromilling has made it possible to convert microfluidic designs to working prototypes more efficiently. CNC milling machines make use of computer aided design (CAD), computer aided manufacturing (CAM) and other software packages to control the milling process resulting in an automated fabrication procedure with features of microscale precision. Overall, micromilling offers a continuous and automated fabrication method with great design flexibility, allowing the rapid delivery of high quality devices with complex designs that might be difficult to achieve with other fabrication methods.

While thermoplastic polymers represent an attractive material of choice for fabricating microfluidic systems, several challenges remain when it comes to utilising these polymers. For one, most of the fabrication methods for thermoplastic-based devices can affect the surface quality of the finished device^22,23^. Surface defects and high surface roughness are common after micromilling due to the process of continuous etching and removal of thermoplastic material by endmills. This would affect the operation, resolution and control of the manufactured microfluidic model^24^. Surface roughness could also impact biological components in the device as cells may orient themselves to grow along the track of the milling tool^20^. During the milling procedure the surface quality can be improved by adjusting the spindle speed and feed rate of the milling tool or by decreasing the step over distance during the milling process^21^. Surface treatment with well-known plasticisers such as cyclohexane, chloroform and acetone can reduce the roughness of micromachined substrates^25–27^. Ogilvie et al.^26^ showed that exposure to chloroform vapour can significantly enhance the surface quality of micromilled PMMA and COC substrates. However, uncontrolled exposure for extended periods of times to chloroform vapour can cause crazing to the surface of microchannels and as such can damage the structure of these channels.

Another challenge when using thermoplastics is their assembly and bonding. Different approaches have been explored to ensure effective and leak-free bonding of thermoplastic-based microfluidic devices. These include thermal bonding, solvent-assisted bonding, adhesive bonding, oxygen plasma treatment and UV-mediated bonding^27–31^. However, several factors should be considered when selecting the appropriate approach for bonding microfluidic chips. For instance, the bonding method should preserve the integrity of the milled microstructures. During thermal bonding, PMMA is heated to a temperature, between 95 and 160 °C, to achieve leak free bonding, which is near or above the glass transitional temperature (Tg) of PMMA 110 °C^32–34^. The use of such high temperatures and pressure can change the mechanical properties of the plastic and consequently can distort the structure of the device leading to deformation of the fabricated structures^35^. Channel clogging and collapse are also common with solvent-assisted bonding, which employs solvents such as ethanol, isopropyl alcohol, 1,2 dichloroethane, chloroform and acetic acid^36^. Bonding of the two thermoplastics can subsequently be achieved by applying a low temperature and pressure for short periods of time. These solvents can impact the quality of the device resulting in structural damage and breakages, especially when an excess of solvent is used. Solvent-assisted bonding can also affect the optical clarity of the finished product. Therefore, when selecting an assembly method, it is important to assess the impact on surface properties, integrity of channels and microstructures, and optical clarity of the microfluidic device in addition to the bonding strength and preservation over time.

Here, we report a versatile method to develop PMMA-based microfluidic devices using micromilling as a microfabrication technique and chloroform-vapour treatment that integrates surface treatment and bonding. This manuscript provides a detailed description of the effect of surface treatment with chloroform vapour on the surface quality and structural integrity of micromilled channels in PMMA. The use of chloroform vapour addresses the limitation of micromilling on the surface quality of finished products as well as providing an excellent bonding method. We employed this method to fabricate a cancer spheroid-on-a-chip model, intended for the long-term culture of multicellular tumour spheroids. Our microfluidic chip system provides the opportunity to recreate important parameters of the tumour microenvironment, such as hypoxia, cellular proliferation and tumour cell quiescence in dynamic culture conditions.

## Results

### Surface properties and roughness

**Figure 1:**
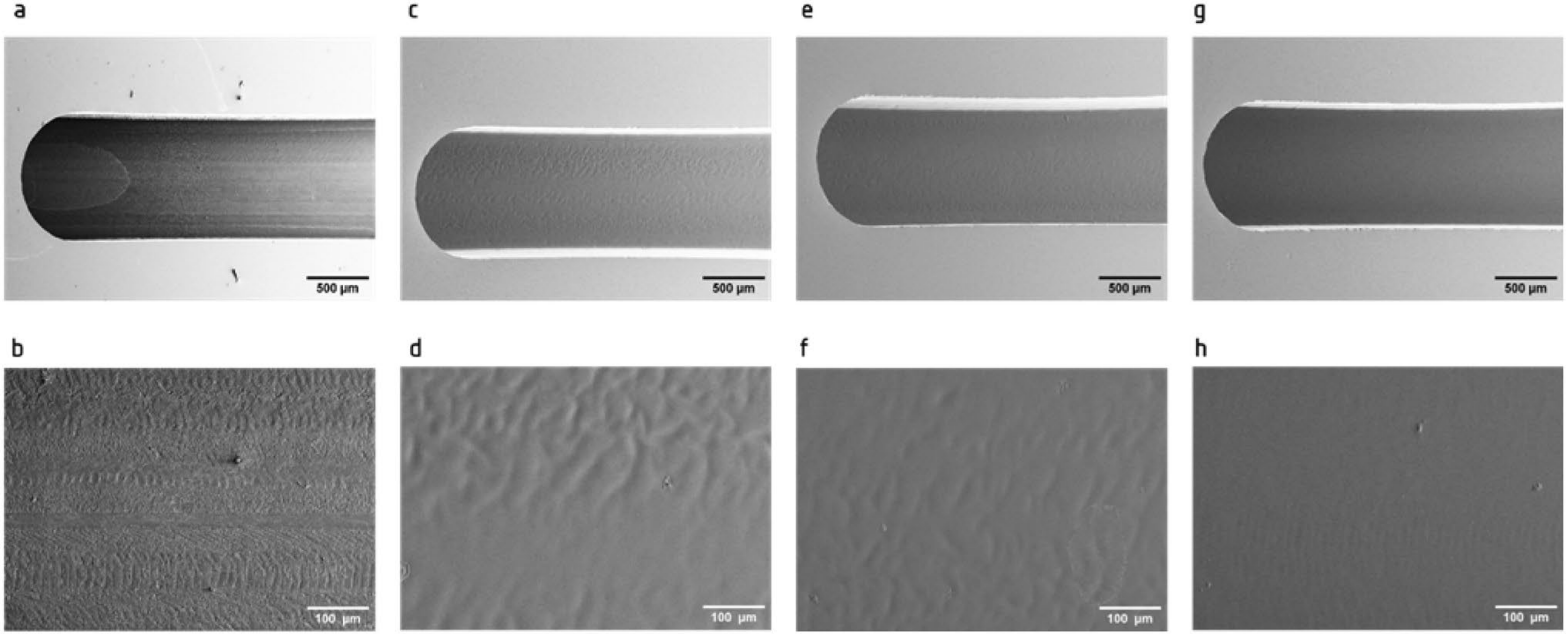
Surface treatment of micromilled PMMA substrates with chloroform vapour. Representative SEM images of micromilled channels after treatment with chloroform vapour for 0 s (**a** and **b**), 60 s (**c** and **d**), 45 s (**e** and **f**) and 30 s (**g** and **h**). (**b**, **d**, **f** and **h**). represent higher magnifications.

While micromilling provides a method for rapid fabrication of microfluidic chips, this approach can generate devices with poor surface quality. Among the different approaches that can improve the surface quality of micromilled substrates, chemical treatment with chloroform vapour may reduce the surface roughness of micromilled PMMA chips. Here, we set out to assess the effect of chloroform vapour on the channel surface quality. First, a channel of 20 mm × 1 mm × 1 mm (l × w × h) was micromilled in PMMA and characterized using SEM. Figure 1a,b show the immediate surface quality of micromilled channels in PMMA after the milling procedure. Traces of the cutting tool and surface defects and indentations were visible on the channel surface (magnification in Fig. 1b). In order to improve the surface quality, micromilled PMMA substrates were exposed to chloroform vapour with increasing exposure times in a custom-built vapour chamber (supplementary Fig. 1a). SEM imaging showed that while treatment with chloroform vapour reduced the tool marks on the surface and improved the surface finish after the milling procedure, longer exposure time altered the surface of the material and the topography of the channel (Fig. 1c,d). Swelling and distortion of the channel surface were visible after 60 s of exposure Fig. 1c, with crevasses also observed as shown in Fig. 1d. Similarly, an exposure time of 45 s remodelled the channel bed, with distortions also visible throughout the channel surface (Fig. 1e,f). In contrast, no distortions of the channel surface were observed after 30 s of chloroform vapour. The treatment enhanced the surface quality of micromilled PMMA channels while maintaining the integrity of the channel surface, as demonstrated in Fig. 1g. The micromilled channel retained its structure and no alteration of their overall shape was observed. The use of chloroform vapour for 30 s provided a reliable and reproducible approach for reducing surface roughness of micromilled PMMA substrates, as shown in Fig. 1h.

**Figure 2:**
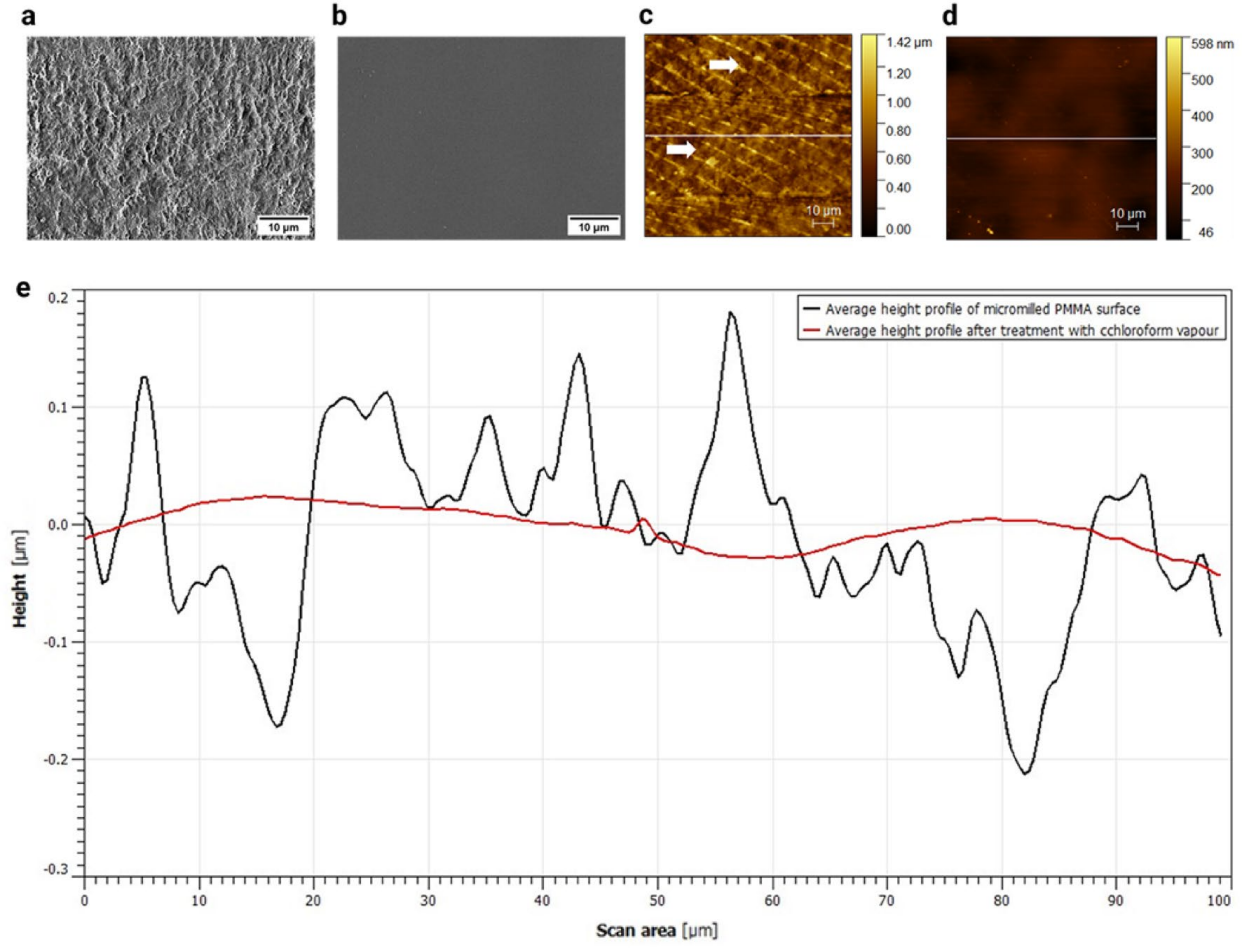
The effect of a 30-s chloroform treatment on the surface of micromilled PMMA thermoplastics. Magnification of representative SEM images of the surface roughness of a micromilled PMMA surface before (**a**) and after (**b**) treatment with chloroform vapour. Representative AFM analysis of surface topography of a micromilled PMMA surface (**c**) before treatment with chloroform vapour, white arrows show the milling tracks on the surface, and (**d**) after treatment with chloroform vapour. The average height profile extracted from micromilled PMMA channels, black line and the average height profile after treatment with chloroform vapour, red line (**e**), n = 3.

A more detailed SEM image analysis showed that the surface of micromilled PMMA initially exhibited poor surface quality with high roughness as the micromilling process had impacted the surface integrity of PMMA (Fig. 2a). Surface chipping and signs of brittle fractures could be observed on the channel surface. After treatment with chloroform vapour for 30 s, a clear, smoothing effect was observed on the surface of micromilled PMMA (Fig. 2b). Chloroform vapour remodelled the surface, with defects removed, and a smoother surface was observed. Subsequently, to assess the impact of chloroform vapour on the surface of micromilled PMMA substrates, AFM measurements of surface topography were conducted. The Root-mean-square (Rq) surface roughness parameter, using Gwyddion, was employed to quantify the impact of a 30-s exposure to chloroform vapour on the surface roughness of micromilled channels. A 20 mm × 10 mm × 1 mm (l × w × h) channel was micromilled in PMMA and the effect of chloroform vapour on the surface roughness and topography was studied across an area of 100 µm^2^. Figure 2c illustrates the surface topography of the micromilled PMMA channel after the milling process, showing a diverse topography with trace of the milling tool and periodic spike in height, indicated by the white arrows. These variations in height may have been caused by the tool moving continuously over the PMMA surface. The Rq roughness of the micromilled PMMA channel was found to be 153 ± 29 nm (mean ± SD, n = 3). Following treatment with chloroform vapour, the surface topography of the micromilled PMMA showed noticeable improvement (Fig. 2d) in comparison to the untreated surface, and significantly reduced the surface roughness to 39 ± 13 nm (mean ± SD, n = 3). This reduction in surface roughness would improve the suitability of micromilled microfluidic devices in biological applications. Previous studies have suggested that high surface roughness might occasionally cause cells to align along the milling tracks (supplementary Fig. 2). Furthermore, the average height profile of multiple chloroform-treated PMMA substrates revealed that chloroform consistently produced a smoother and more homogeneous surface compared to the average height extracted from micromilled surfaces, n = 3, as shown in Fig. 2e. Overall, these findings demonstrate that controlled exposure to chloroform for 30 s can generate a more uniform and smoother surface, while preserving the integrity of the channel structure.

### Chloroform vapour-assisted thermal bonding

With the growing interest in thermoplastics as a base material for microfluidic chips, the need for a simple, reliable and non-destructive bonding method for chip assembly is essential. As demonstrated above, controlled exposure to chloroform vapour improves the surface roughness of micromilled PMMA substrates and conserves the structures engraved. In general, chloroform can also act as a plasticiser that reduces the Tg of PMMA, which provides a bonding procedure that can be performed at a relatively low temperature and pressure. Consequently, we sought to combine the surface effect of chloroform treatment while providing a strong and non-destructive bonding approach. The bonding strength was evaluated using a standard tensile strength test, and was compared with a commonly used bonding method, namely solvent-assisted bonding with 70 and 90% ethanol. PMMA substrates were bonded over an area of 4 cm^2^ and the bonding strength was characterised by determining the mechanical stress required for the two PMMA substrates to detach (supplementary Fig. 3). Results showed that treatment with chloroform vapour, resulted in an irreversible bond where PMMA substrates were permanently attached and could only be detached through destruction of the overlapping area. The tensile strength of the bonding was measured to be 9.07 ± 0.9 MPa (mean ± SD, n = 3) after 24 h of bonding (Table 1). This bonding strength was higher than the bonding strength of 70 and 90% ethanol, i.e., at 7.62 ± 0.6 and 7.88 ± 0.9 MPa (mean ± SD, n = 3), respectively. To further examine the stability and durability of this bonding method, we analysed the bonding strength one month after bonding, with PMMA substrates being stored at room temperature. The tensile strength of chloroform-bonded samples was 9.11 ± 0.5 MPa (mean ± SD, n = 3), similar to the strength obtained immediately after 24 h. This indicates that the bonding did not weaken over time. Furthermore, the bonding strength remained significantly higher than after bonding with ethanol, where 6.92 ± 0.3 and 6.98 ± 0.3 MPa (mean ± SD, n = 3), was achieved with 70% and 90% ethanol, respectively (Table 1). These results show that vapour-assisted bonding with chloroform can provide strong, stable bonding and an attractive option for long term usage of PMMA-based microfluidic devices. Furthermore, chloroform vapour-assisted bonding does not affect the integrity of the structures micromilled in PMMA (supplementary Fig. 4).

**Table 1:** Tensile strength of vapour- or solvent-assisted thermal bonding of PMMA, n = 3.

Bonding method	Bonding strength after day 1 (MPa, mean ± SD)	Bonding strength after month 1 (MPa, mean ± SD)
Vapour treatment with CHCl_3_	9.07 ± 0.9	9.11 ± 0.5
Solvent treatment with 90% Ethanol	7.88 ± 0.9	6.98 ± 0.3
Solvent treatment with 70% Ethanol	7.62 ± 0.6	6.92 ± 0.3

### Microfluidic spheroid-on-a-chip device

**Figure 3:**
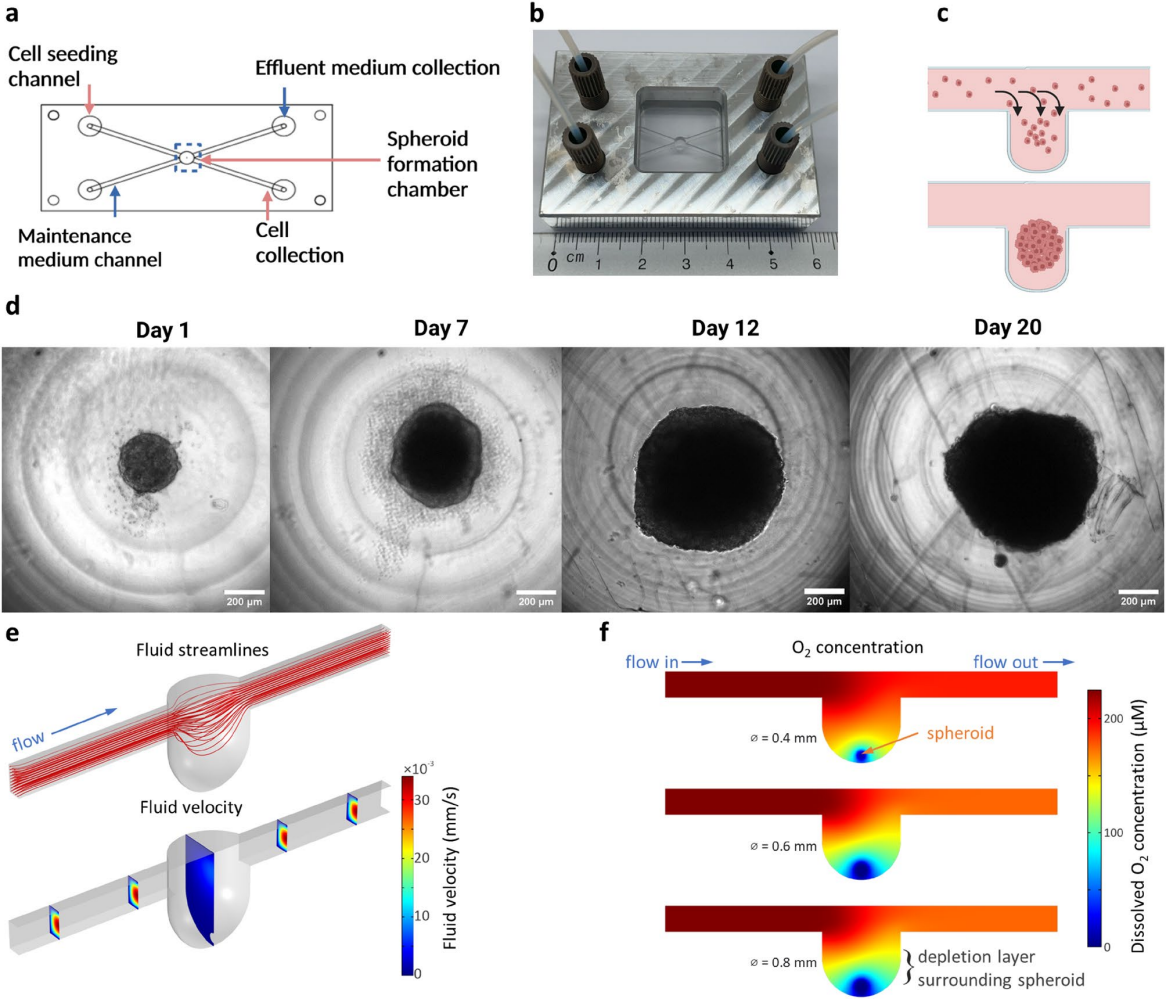
PMMA-based spheroid-on-chip design and operation. Design of the spheroid-on-a-chip device, micromilled in PMMA (**a**). Assembled system, consisting of the PMMA chip inserted into an aluminium holder, and tubing connected via HPLC connectors (**b**). Once introduced the microfluidic device, spheroid formation is based on self-assembly of entrapped cells inside a repellent microwell (**c**). MDA-MB-231 multicellular tumour spheroid growth was observed over the course of 20 days (**d**). Simulation showing the streamlines and fluid velocity in the microwell and connecting microchannels (symmetric domain) (**e**). Simulated steady-state contours of dissolved oxygen within the region surrounding each microwell for three different spheroid sizes (0.4, 0.6, and 0.8 mm diameter) (**f**).

Subsequently, we fabricated a cancer spheroid-on-a-chip device, using the optimised procedures described above. Spheroids represent an attractive model to study a rudimentary tumour microenvironment (TME). We aimed for this device to support long-term culture of multicellular tumour spheroids to highlight the potential of PMMA, and this rapid fabrication and assembly method for durable microfluidic devices. The chip design is provided in Fig. 3a. The chip was micromilled from two layers of PMMA. The bottom layer featured a concave shaped microwell with a diameter of 3 mm and a depth of 2.5 mm, to allow the formation and maintenance of spheroids. The microwell was connected to a channel, through which tumour cells were introduced into the device. This channel, 1 × 1 mm (w × h), allowed for rapid seeding and cell entrapment inside the microwell. The upper layer of the device consisted of two inlets and two outlets as well as an additional channel. This upper channel was designed to supply medium, with oxygen and nutrients to the spheroid once it had formed and remove waste that accumulated in the microwell. Furthermore, a metal holder was designed to secure the PTFE tubing in the chip, allowing the two inlets and outlets to use microfluidic connectors (ferrule and nut, Fig. 3b). The formation of 3D spheroids in this microfluidic device was based on the self-assembly of the entrapped tumour cells inside the microwell (Fig. 3c). This well was made cell repellent by coating with pluronic F-127 before seeding, to promote tumour cells to aggregate and form 3D spheroids. Via syringe pumps, MDA-MB-231 tumour cells were introduced into the microfluidic chip in regular medium supplemented with a small amount of Matrigel at a cellular density of 1 × 10^5^ cells/mL and a flow rate of 30 µL/minute for 15 min. After the initial seeding flow, regular medium with Matrigel was flushed through the channel at a flow rate of 20 µL/min for another 15 min to remove excess cells that were trapped inside the channels. This step prevented the formation of small cellular aggregates that could interfere with the spheroid that was cultured inside the microwell. Cells trapped in the seeding channel had the ability to change the shape of the spheroid and subsequently non-uniform spheroids would form over time (supplementary Fig. 5).

After seeding, MDA-MB-231 cells formed a clear spherical structure within 24 h of culture (see supplementary Movie 1). The newly formed spheroid was incubated for a further 24 h, during which it formed a more compact structure, before maintenance flow at 1 µL/minute was initiated through the top channel. Continuous observation and bright field imaging showed that MDA-MB-231 spheroids continued to grow over time, with an increase in diameter. After 24 h of culture the diameter was approximately 398 ± 40 µm (mean ± SD, n = 7), which increased to 727 ± 62 µm (mean ± SD, n = 7) after 12 days in culture (Fig. 3d). The formed spheroids remained viable for at least 20 days inside the microfluidic chip, as was evident by the compact structure, well-defined perimeter and the absence of cellular debris around the spheroid (Fig. 3d). This shows that long-term culture of tumour spheroids is possible within this microfluidic design. Numerical simulations of fluid flow show a large drop (~ 10 ×) in fluid velocity in the microwell when compared to the maintenance microchannels, allowing for sufficient nutrient delivery with reduced viscous shear (Fig. 3e). The methods described by Grimes et al*.* were used to estimate the rate of oxygen consumption in spheroids of varying diameter situated at the bottom of a microwell^37^. Simulations of dissolved oxygen transport revealed a spherical-like depletion layer in the direct vicinity of each spheroid, where the total oxygen consumption was less than 25% of that provided with the input flow, where respective nutrient (glucose) consumption was less than 0.3% (Fig. 3f, supplementary Fig. 6, 7 and 8). These results show that long-term culture of tumour spheroids is possible within this microfluidic design with continuous supply of oxygen and nutrients.

### Proliferation, cellular quiescence and hypoxia

**Figure 4:**
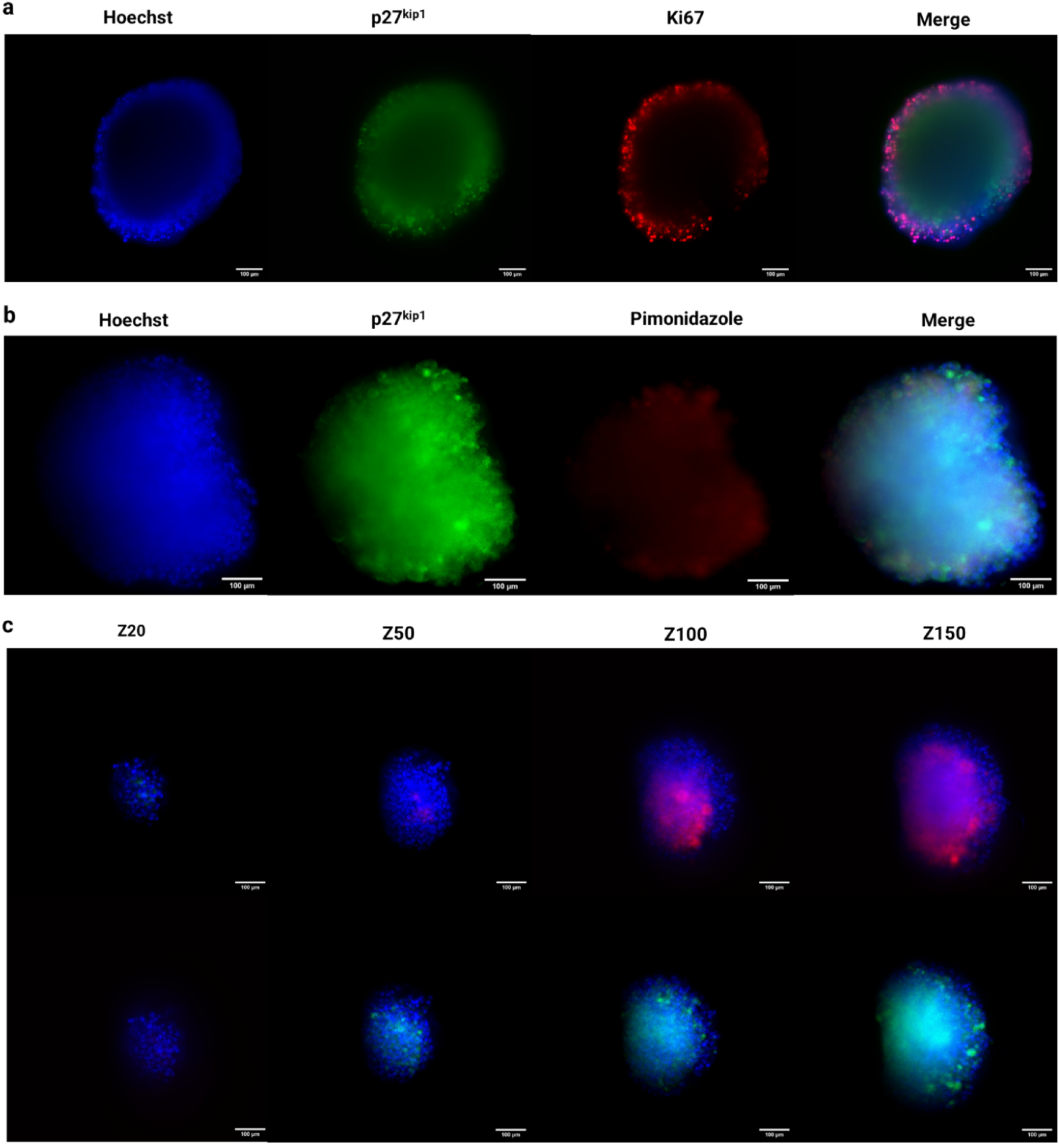
(**a**) Immunofluorescent staining of MDA-MB-231 spheroids with the proliferation marker Ki67 (Red), quiescent marker p27^kip1^ (Green) and the nuclear stain Hoechst (Blue). (**b**) Immunofluorescent staining of MDA-MB-231 spheroids with quiescent marker p27^kip1^ (Green) and the hypoxia marker pimonidazole (Red) and the nuclear stain Hoechst (Blue). (**c**) Montage of subset of acquired z-planes at 1 µm intervals. Red is pimonidazole, green is p27^kip1^ and blue is Hoechst.

Next, we performed immunostainings to characterize the proliferative heterogeneity and presence of hypoxia in the MDA-MB-231 spheroids cultured in this microfluidic model. In general, multicellular tumour spheroids display many characteristics found in the TME, such as gradients in oxygen, nutrients, and cell proliferation, the latter accompanied by a quiescent region. First, we examined the presence of the proliferation gradient by staining the spheroid with the proliferation marker Ki67 and the quiescence marker p27^kip1^. Ki67 is a marker of cell proliferation, widely used to evaluate the proliferation index in tissue biopsies, while p27^kip1^ is a marker of cellular quiescence and cell cycle arrest^38,39^. Analysis of these markers in 12-day old spheroids are shown in Fig. 4. Results revealed that positive expression of Ki67 was mostly detected in tumour cells that reside in the outer rims of the spheroid, whereas cell populations toward the spheroid core showed no noteworthy staining (Fig. 4a). In contrast to Ki67, tumour cells that were closer to the core showed expression of p27^kip1^. These results indicate that spheroids cultured inside our microfluidic device exhibit the characteristic proliferation gradient that is present in tumour spheroids cultured in standard well plates under static conditions.

Next, staining for hypoxia using pimonidazole-labelling showed that MDA-MB-231 spheroids exhibited a clear hypoxic core after 12 days of culture. Furthermore, the proliferative status of the cellular population present within this hypoxic core was investigated using the quiescent marker p27^kip1^ (Fig. 4b). We detected pimonidazole staining in the core of our MDA-MB-231 spheroids. Additional staining with p27^kip1^ revealed that cells within these hypoxic regions are quiescent (Fig. 4b). Using a light sheet microscope, different Z-stack images were taken at intervals of 1 µm. Analysis of Z-stack images revealed that MDA-MB-231 spheroids exhibit a hypoxic core, which was detected 50 µm from the surface of the spheroid (Fig. 4c). This observation aligns with previous findings^40^.Similarly, Z-stack images confirmed that the cell population inside these low oxygen regions expressed p27^kip1^, indicating the presence of quiescent cells. Both hypoxia and quiescence became more prominent deeper inside the spheroid. These observations indicate that multicellular spheroids cultured within this microfluidic device recreate the proliferation and oxygen gradient typically observed in human solid tumours in vivo. Therefore, our microfluidic system provides a robust biological model to study the physicochemical gradients of the TME and their effects on tumour cell behaviour in dynamic conditions.

## Discussion

With their favourable characteristics and the plethora of microfabrication techniques available, thermoplastic polymers represent an attractive option for microfluidic devices in research areas where the choice of material is imperative. Here, we described a method for rapid fabrication and assembly of PMMA-based microfluidic devices using micromilling and chloroform vapour-assisted bonding. The surface of structures micromilled in slabs of PMMA exhibited substantial roughness when left untreated. We showed that treatment with chloroform vapour smoothed the surface and could simultaneously be used as a method to generate strong and durable bonding of multi-layer microfluidic chip systems. We demonstrated the functionality of this method with a purpose-built tumour spheroid-on-a-chip device. This device allowed culture of multicellular tumour spheroids under flow for at least three weeks. Furthermore, analysis of the spheroids revealed the presence of the characteristic proliferative, quiescent and hypoxic regions.

Micromilling provides a versatile and rapid production method for microfluidic devices, offering an automated approach that can directly generate both simple and complex designs with high precision and resolution (~ 20 μm)^41–43^. The ability to convert a design from a 3D modelling program to a working prototype is one of the most appealing features of this fabrication approach and makes it one of the most desirable methods to rapidly develop microfluidic devices. However, as a subtractive method it can impact the quality of the micromilled chip and affect the potential applications of the device. For instance, if optical clarity is essential, poor surface quality can impact image acquisition and microscopy. Or, if surface interactions between other (biological) materials and the device are crucial for chip function, high surface roughness would undermine the performance of the device^44^. Following micromilling of microchannels in PMMA substrates, we investigated the surface finish using SEM and AFM. SEM revealed that the milling process affected the surface quality as milling tracks and marks from the endmill tool were visible on the channel surface. AFM analysis showed that the milling procedure produced a surface of varying topography and with grooves along the surface of the channel due to the fabrication procedure. We showed that this roughness can be mitigated by surface treatment with chloroform vapour. It was previously reported that the use of chloroform, cyclohexane, and acetone for chemical polishing is an effective method to reduce the surface roughness of various machined thermoplastic materials^25,26,45,46^. After exposure to chloroform vapour, we also observed a clear improvement in the surface quality of the micromilled channel. Consistent with earlier research that employed solvent vapour treatment to improve the surface finish of PMMA microfluidic devices, we have demonstrated that precise control of chloroform exposure times is essential to ensure that the micromilled structures on PMMA remain intact^27,46^. Long exposure times to chloroform vapour resulted in cracking and deformation of the channel bed, affecting the integrity of microstructures in thermoplastics. However, a 30-s exposure to chloroform vapour improved the channel surface, producing a more homogenous surface topography with reduced surface roughness, without affecting the integrity of the channel. Previous research highlighted that surface roughness can affect cell behaviour, implying that poor surface quality may compromise the biocompatibility of machined microfluidic devices^47^. Nevertheless, studies have demonstrated only minimal impact on the biological performance of micromilled devices when the surface roughness was approximately 0.45 µm^20^. In our hands, treating the surface with chloroform vapour reduced the roughness to around 0.03 µm, suggesting that we diminished the roughness to a sufficient level for biological applications.

While surface treatment with chloroform vapour offers a method to reduce surface roughness, effects may differ depending on the nature and shape of structures micromilled in PMMA. The effects of chloroform vapour on surface quality as reported previously were mostly observed on microchannels with flat surfaces. Consequently, treatment with chloroform vapour may need to be adjusted for more intricate structures with concave and circular surfaces. For biological application, cells may be seeded on flat surfaces and microchannels, which may necessitate surface smoothing. Another challenge with this treatment is that the effect of chloroform vapour on the autofluorescence of PMMA remains unclear. In general, PMMA has the same levels of autofluorescence of glass of the same thickness, however chloroform can change the polymer crosslinking on the surface, which may increase the autofluorescence of PMMA substrates. We also explored chloroform vapour as an effective approach for bonding PMMA-based microfluidic devices^48^. This approach was inspired by previous work from Ogilvie et al*.* who exposed PMMA substrates to chloroform vapour for 4 min before initiating the bonding process^26^. Their bonding procedure was carried out at relatively low temperature and pressure due to the effect of chloroform vapour on the surface of PMMA^26^. In our study we sought to combine the effect smoothing effects of chloroform vapour PMMA with a robust high throughput and reliable bonding method. Following exposure to chloroform vapour for 30 s at 30 °C, PMMA substrates were bonded at 65 °C and the bonding strength was analysed using a standard tensile test. The tensile strength achieved was higher than the strength through solvent assisted bonding using 70 or 90% ethanol. Furthermore, the bonding was stable when samples were stored for one month. Bonded substrates showed a tensile strength similar to that obtained immediately after the bonding procedure, indicating that this approach can provide a stable and durable bond. Overall, our work shares an optimized procedure for others to generate a solid and robust foundation for PMMA-based microfluidic devices, for example for application in organ-on-a-chip systems. This approach also reduces the destructive effects normally observed with thermal bonding on channels or caused by excessive solvents during solvent bonding.

We have demonstrated the applicability of this fabrication and assembly method in the development of a tumour spheroid-on-a-chip device. Multicellular tumour spheroids have garnered great interest in cancer research as a biological model that can replicate important aspects of the TME, such as proliferation gradients accompanied with quiescent zones, but also nutrient and oxygen gradients^49^. Combining these features with microfluidic systems could generate an advantageous in vitro model system that can faithfully reproduce the physicochemical gradients of the TME in conditions that are highly dynamic. As a rapid procedure for development and fabrication of microfluidic devices, micromilling has made the fabrication of this model system relatively straightforward. Challenges encountered when using PDMS-based microfluidic devices, namely the need for a dedicated clean room or extensive fabrication steps, can be overcome. Furthermore, the use of PMMA as an alternative material addresses the fundamental limitations of PDMS and provides a device that can potentially support long term culture without the need for further treatments or surface modifications. Here, we have demonstrated a simple and user-friendly approach for generating an efficient system to culture multicellular spheroids for biomedical research.

In our microfluidic set-up, tumour cells were seeded in non-adherent microwells forcing them to form aggregates, as described previously^40^. Tumour cells were introduced into the microfluidic chip using syringe pumps. The simple design of the chip allowed for the entrapment of tumour cells inside cell-repellent microwells, where they formed spheroids. MDA-MB-231 breast cancer cells were capable of self-assembling into multicellular spheroids within 24 h of seeding. This standardised approach eliminates the need to culture spheroids outside the device before their incorporation into microfluidic models, and allow better control over spheroid size^50–53^. Our model system shows that multicellular spheroids cultured within this system are highly reproducible as they exhibit uniform size and a distinct spherical structure. Our spheroids continued to grow over time, and remained viable as indicated by their compact structure and clear borders. After 12 days, tumour spheroid size was measured at 727 µm. Notably, previous microfluidic platforms for culturing multicellular spheroids showed that spheroids can grow to a maximum of 250 µm. These models focus on the production of high numbers of spheroids for high throughput screening of chemotherapeutic agents^51,54,55^. However, it has been demonstrated that spheroids with a size of 500–600 µm can develop proliferation gradients that resemble the in vivo situation. These gradients consist of a proliferative layer near angiogenesis-induced capillaries, a quiescent intermediate layer, and an inactive necrotic core that lies further from blood supply^56,57^. We sought to develop a microfluidic system that can accurately reproduce these gradients and support long-term culture and manipulation under various conditions. Furthermore, the dynamic nature of this device ensures that multicellular spheroids cultured have continuous supply of oxygen and nutrients during experiments.

We performed immunofluorescent staining to characterize our MDA-MB-231 spheroids cultured within this microfluidic system. Results from this experiment showed that MDA-MB-231 spheroids exhibited a clear hypoxic core, as was reported previously^40,58^. Dual staining with the quiescent marker, p27^kip1^, indicated that tumour cells within this hypoxic core are mostly quiescent. Therefore, it could be suggested that multicellular tumour spheroids generated within this microfluidic device can—in part—recreate the complex TME in vitro providing the opportunity to evaluate chemotherapeutic agents in a more physiologically relevant biological platform. Furthermore, MDA-MB-231 spheroids cultured within our microfluidic chip remained viable for at least 20 days of culture. The dynamic nature of the microfluidic system ensured a continuous supply of nutrients. As such, we were able to support multicellular spheroids for long-term culture while retaining the physicochemical gradients present in vivo. This model system offers a platform that can assess the effects of the TME on tumour cell behaviour, proliferation and metastasis. Particularly, it has the potential to investigate long-term processes, such as drug exposure and observation of chemotherapeutic activity with repeated exposure on tumour cell response, highlighting chemotherapeutic uptake, distribution and pharmacokinetic parameters. Furthermore, our design facilitates the retrieval and handling of multicellular spheroids by gently flushing them at a low flow rate through the channels, allowing for further analysis and quantitative studies of spheroids cultured under dynamic conditions. Previous spheroid-on-a-chip microfluidic devices have been limited in their ability to retrieve spheroids, either due to the chip design or the damage incurred during the retrieval process^59^. Additionally, the optical clarity and transparency of PMMA enable on-chip monitoring and analysis using optical and fluorescent microscopy. The use of PMMA also addresses a key challenge in microfluidic systems, as most microfluidic devices are designed for single-use only, and the high cost of microfabrication processes and labour has limited their widespread use for drug screening. PMMA has low protein binding efficiency and low sensitivity, making it easier to clean and remove proteins and other biological materials from surfaces. This allows for multiple usage of microfluidic systems after appropriate washing and sterilization steps. Furthermore, rapid fabrication methods can enable the quick and affordable production of complex 3D microfluidic devices with integrated components, increasing their efficiency for high-throughput screening.

Though our current microfluidic model is limited to culturing one spheroid per chip, further iterations of this design could include an array to seed and culture multiple spheroids per chip. As such, the system would streamline higher throughput chemotherapeutic screening, while recreating the physicochemical gradients present in vivo. It is important to note that our current microfluidic model does not take into account the transport of molecules through the physiological barriers present in the human body. This could be achieved by incorporating commercially available synthetic membranes or by adding an endothelial layer to simulate drug transport. Furthermore, the simple design of this microfluidic system enables the introduction of stromal cell types including cancer-associated fibroblasts to better represent the different cellular populations present within the TME. This approach would enable researchers to understand the links between the heterogenous microenvironment and cancer biology by recreating the different mechanical, physical and chemical cues present with the TME.

## Conclusion

The compatibility of the material interface in microfluidic devices must be carefully evaluated for each application, as the suitability for one application may not extend to another. Moreover, the complexity of microfabrication techniques, coupled with the need for specialized facilities, can make the process labour-intensive and difficult to implement. Thermoplastic polymers are emerging as a suitable material for developing microfluidic devices due to their mechanical and chemical properties and biocompatibility, in addition to the vast array of microfabrication techniques available. Here, we presented a method for fabrication and assembly of PMMA-based microfluidic devices using micromilling and chloroform-vapour treatment. We have shown that micromilling can provide a rapid fabrication approach, capable of developing microfluidic systems with a high degree of complexity and flexibility. The use of chloroform vapour very effectively reduced the surface roughness of micromilled channels and provided an effective method for bonding PMMA-based microfluidic devices. This approach maintained the structures engraved on PMMA while providing a strong and stable bond that was durable for long periods of times. The accessibility of this fabrication approach was demonstrated by the development of a microfluidic spheroid-on-a-chip device. This microfluidic system offers a valuable model for generation, maintenance, and analysis of multicellular tumour spheroids in dynamic culture conditions. While this microfluidic device can support long term culture, multicellular spheroids generated within this device can recreate important physicochemical gradients present within the TME in vivo, making it a valuable tool in cancer research.

## Materials and methods

### Chemicals and reagents

Isopropyl alcohol was acquired from Macron Fine Chemicals (Avantor, Arnhem, The Netherlands; 6775–25), chloroform from Acros organics (Fisher Scientific, The Hague, The Netherlands; 383,760,025). Ethanol and hydrogen peroxide were from Boom B.V. (Meppel, The Netherlands; 84,050,062 and 76,051,800 respectively). Invitrogen UltraPure DNase/RNase-Free Distilled Water was obtained from Fisher Scientific (The Hague, The Netherlands; 12,060,346) and 4% formaldehyde and Triton X-100 from Sigma-Aldrich (Zwijndrecht, The Netherlands; P6148 and T9284, respectively).

### Micromilling

The geometry of microfluidic channels and different microstructures were designed using the 3D modelling software SolidWorks premium 2021 (Dassault Systèmes, Paris, France). A benchtop Minitech CNC Mini-Mill/GX micromilling machine (Norcross, GA, USA) was used to mill microstructures directly into 3 mm thick PMMA sheets (Extruded Plexiglas® acquired from Colltec, Groningen, The Netherlands). The micromilling set-up used SolidWorks CAM (Dassault Systèmes, Paris, France) and Mach3 software (Newfangled Solutions LLC, Livermore Falls, ME, USA) to generate G-code commands that controlled the milling tool in terms of feed rate and spindle speed to fabricate and create structures in PMMA. A combination of milling tools (Ceratizit, Mamer, Luxembourg) were used to engrave channels and microstructures in PMMA.

### Scanning electron microscopy (SEM)

Micromilled PMMA substrates containing 20 mm × 1 mm × 1 mm (l × w × h) channels were thoroughly cleaned with milli-Q water in an ultrasonic bath. Next, the substrates were rinsed with isopropyl alcohol and dried with nitrogen gas to remove any debris remaining from the milling process. PMMA substrates were uniformly exposed to chloroform vapour in a custom-built vapour reservoir, where chloroform was preheated for 10 min at 30 °C (supplementary Fig. 1a). PMMA substrates were positioned on metal pillars on top of the reservoir, with the micromilled channels facing the chloroform. The channels were exposed to chloroform vapour for 30, 45 or 60 s. The effect of chloroform vapour on the surface properties of micromilled channels was imaged using a Supra 55 Scanning Electron Microscope (Jena, Germany).

### Atomic force microscopy (AFM)

A channel, 20 mm × 10 mm × 1 mm (l x w x h), was micromilled in PMMA. The chip was treated with chloroform vapour for 30 s and the surface topography of the channel was subsequently analysed using AFM (Nanoscope V, Veeco Instrument, Santa Barbara, CA, USA). Surface scans and topographic images were obtained with the AFM Dimension 3100 Nanoscope V system operating in tapping mode in air (model: DNP-10). To determine the surface topographies, the obtained AFM images were analysed using Gwyddion Analysis software. The surface roughness was analysed by using the parameter Root-mean-square roughness (Rq). Rq was used due to its sensitivity to surface peaks and valleys, making it an accurate measure of surface quality. To obtain more representative results, three measurements were collected per sample from three samples per condition.

### Chloroform vapour-assisted thermal bonding

Prior to the start of the bonding procedure, micro-milled PMMA chips, 50 mm × 20 mm × 3 mm (l × w × h), were thoroughly cleaned as described above and treated with chloroform vapour for 30 s. Chips were assembled immediately and aligned together with the coordinated inlets and outlets, and placed inside a hot embossing machine (Collin Lab & Pilot Solutions GmbH, Maitenbeth, Germany) at 65 °C for 20 min. The bonded devices were then gradually cooled to 45 °C and then 25 °C in 15 min. The entire bonding procedure was conducted at a pressure of 14 bar. The bonded chip was allowed to rest overnight at room temperature to ensure the evaporation of any chloroform that might be trapped in the channels.

### Bond strength testing

To test the bonding strength, standard PMMA substrates of 50 mm × 20 mm × 3 mm (l × w × h) were cleaned as described above. The substrates were partially bonded over an area of 4 cm^2^ using chloroform vapour as described above (supplementary Fig. 3). PMMA substrates were also bonded using two different concentrations of ethanol, namely 70 and 90%. Ethanol was added to the surface of the PMMA substrates, which were then pressed together using metal clamps and incubated at 75 °C for 10 min. PMMA substrates were cooled to room temperature over the period of 1 h. The tensile testing was carried out using an Instron 4301 universal testing machine (Instron, High Wycombe, UK) with a 5 kN load cell at a cross-head speed of 2 mm/min. The tensile force required for the overlap bonded area of PMMA to break was recorded and was divided by the cross-sectional area to obtain the tensile strength of adhesion (supplementary Fig. 3).

### Spheroid-on-chip fabrication and assembly

A microfluidic chip for culturing multicellular tumour spheroids was designed and milled on standard PMMA substrates. The channels were micromilled by employing a 0.5 mm flat micromilling tool (free length 2.5 mm : Ceratizit, 52,802,054) and a 0.5 mm flat micromilling tool (free length 5 mm : Ceratizit, 52,802,055). The spheroid chamber was fabricated using a micro ball nosed with a 0.8 mm cutting diameter and free length of 4.0 mm and a ball nosed radius of 30° (Ceratizit, 52,804,084). Prior to bonding, the two PMMA parts were cleaned as described above. The device was bonded using chloroform vapour-assisted thermal bonding, as described above. Briefly, the two surfaces to be bonded were treated with chloroform for 30 s, then brought together using an alignment tool (supplementary Fig. 1b), and placed inside the hot embossing machine for 20 min at 65 °C. The device was allowed to cool gradually to 45 °C and then 25 °C in 15 min. The chip was sterilised with 3% (v/v) hydrogen peroxide in water for 30 min and flushed with distilled water to remove residual hydrogen peroxide from the channels. Prior to culturing spheroids, the chip was treated with 5% (w/v) pluronic-F127 (Sigma-Aldrich, P2443) in distilled water and left overnight at room temperature.

### Cell culture

MDA-MB-231 cells were obtained from LGC promochem (London, UK) and maintained in high glucose Dulbecco’s Modified Eagle’s medium (DMEM, Gibco, 31966–021) supplemented with 20 mM HEPES buffer solution (1 M, VWR Life Science, J848-500 mL), 1 × MEM Non-Essential Amino Acids (MEM-NEAA, Gibco, 11140–035), 1 × Penicillin/Streptomycin solution (Gibco, 15140–122) and 10% heat-inactivated Foetal Bovine Serum (FBS, Gibco, 10500–064), at 37 °C and 5% CO_2_. Cells were routinely passaged at 90% confluence by washing with Dulbecco’s phosphate-buffered saline (DPBS) and harvesting with 0.05% Trypsin–EDTA (both Gibco, 14190–144 and 25300–104, respectively).

### Spheroid formation and maintenance

Single-use syringes 1, 5 and 20 mL were connected to the microfluidic chip using polytetrafluoroethylene (PTFE) tubing (Polyfluor Plastics B.V., Breda, the Netherlands, 806a08 × 16) and operated via syringe pumps (NE-1000, ProSense B.V., Oosterhout, The Netherlands). Introduction of MDA-MB-231 cells into the chip was performed by perfusing a cell suspension at a flow rate of 30 μL/min for 15 min. Cell culture medium was supplemented with 2.5% (v/v) Matrigel (Corning, 354,234). Subsequently, a flow rate of 20 μL/min was applied for 15 min in the same channel to ensure no cells remained in the channels, thereby reducing the possibility of spheroid formation in the channels. The chip was incubated for 24 h at 37 °C and 5% CO_2_ in static conditions to allow spheroid formation. Next, maintenance medium at a flow rate of 1 μL/min was introduced to the chip in order to supply the newly formed spheroids with nutrients and remove accumulated waste from the spheroid chamber.

### COMSOL simulation

The finite element simulation software COMSOL was used to simulate both fluid flow (Navier–Stokes equations) and nutrient transport (oxygen, glucose; convection–diffusion equations) within a domain that represented the microwell structure and the inlet/outlet microchannels. Simulations were performed under steady-state conditions, where we considered multicellular spheroids of various diameters (0.4–0.8 mm) resting on the bottom of the microwell. Oxygen consumption rates were estimated using the previous methods, and we assumed aerobic respiration with an overabundance of glucose. Further simulation details, including a mesh convergence study, can be found in the supporting information.

### Immunofluorescent staining for proliferation, quiescence and hypoxia

After 12 days of culture, MDA-MB-231 spheroids were collected from the microfluidic chip and stained for proliferation and quiescence. Spheroids were fixed with 4% formaldehyde for 30 min at room temperature. After fixation, they were washed with DPBS and permeabilised with 0.5% (v/v) Triton X-100 in DPBS for 45 min at room temperature. Next, they were incubated overnight at 4 °C with primary antibody against Ki67 (mouse mAb (8D5), 9449S, Cell Signaling Technology, Danvers, MA, USA) that was diluted 1:400 in antibody diluent solution (ABD), consisting of 5% (w/v) BSA (Sigma-Aldrich, A9647) and 0.1% (v/v) Tween-20 (Sigma-Aldrich, P7949) in DPBS. Next, after washing three times with DPBS, spheroids were incubated for 1 h at room temperature with Alexa 555-conjugated goat anti-mouse IgG (Fisher Scientific, A-21422) diluted 1:100 in ABD. After washing with DPBS, the staining proceeded with overnight incubation at 4 °C with the primary antibody against the quiescence marker p27^kip1^ (rabbit mAb (D69C12), 3686 T, Cell Signaling Technology). The antibody was diluted 1:800 in ADB. Following the overnight incubation, spheroids were washed with DPBS to remove any excess antibody and then incubated for 1 h at room temperature with Alexa 488-conjugated donkey anti rabbit IgG (Fisher Scientific, A-21206), diluted 1:100 in ABD. Nuclei were counterstained with Hoechst 33342 (Fisher Scientific, 62249), diluted 1:5,000 in DPBS, overnight at 4 °C to allow better penetration of the dye. Spheroids were collected after staining and were embedded in 1.2% Agarose (Sigma-Aldrich, A6013) and were imaged using a Zeiss LS7 light sheet microscope (Jena, Germany).

MDA-MB-231 spheroids were also stained for the hypoxia marker pimonidazole after 12 days of culture. Briefly, MDA-MB-231 spheroids were incubated with 200 μM pimonidazole hydrochloride (Hypoxyprobe™-1, Burlington, MA, USA) added to the medium for 1 h at 37 °C and 5% CO_2_. MDA-MB-231 spheroids were washed three times with DPBS and fixed with 4% formaldehyde for 30 min at room temperature. After fixation, the spheroids were washed with DPBS and permeabilised with 0.5% (v/v) Triton X-100 in DPBS for 45 min at room temperature. Following permeabilisation, spheroids were washed with DPBS and incubated overnight at 4 °C with IgG1 mouse monoclonal-anti-pimonidazole (Hypoxyprobe, 70132513), diluted 1:100 in ABD. Next, spheroids were collected, washed with DPBS and incubated for 1 h at room temperature with Alexa 555-conjugated goat anti-mouse IgG, diluted for 1:100 ratio in ABD. After washing with DPBS, nuclei were counterstained with Hoechst 33342, diluted 1:5,000 in DPBS overnight at 4 °C. After staining, MDA-MB-231 spheroids were collected, embedded in agarose and imaged with Zeiss LS7 light sheet microscope.

### Image acquisition, microscopy and analysis

In situ, spheroids were imaged within the microfluidic chip using a Leica inverted microscope DM IL3 with a 5X and 10X objective. Live cell imaging was performed using a Cytosmart Lux3FL system (Eindhoven, The Netherlands). ImageJ (National Institute of Health, Bethesda, MD, USA) was used to measure the diameter of MDA-MB-231 spheroids, by converting pixels to µm. The growth of MDA-MB-231 spheroids was tracked in this way. Immunofluorescently stained spheroids were imaged off-chip using a Zeiss LS7 light sheet microscope (Jena, Germany). The images were acquired at the corresponding emission and excitation wavelengths for each fluorophore that was used. Images were analysed using Zeiss Zen Blue edition software (Jena, Germany) and ImageJ. Zen Blue was also used to reconstruct Z-stack images.

### Statistical analysis

Statistical analysis was conducted with GraphPad Prism 8 (GraphPad Software, Inc., CA, USA). Data were generated for a minimum of three repeats and mean values and standard deviation calculated. One-way analysis of variance (ANOVA) and Unpaired t-test were used to perform data statistical analyses. *p* < 0.05 was considered to be statistically significant.

### Ethics declarations

We hereby confirm that this research was conducted with the highest levels of integrity and adherence to ethical guidelines. This study did not involve human participants, human materials, or animal subjects.

### Supplementary Information

Supplementary Figures.

Supplementary Video 1.

## Data Availability

All raw data generated during this study are available from the corresponding author on request.
