# Supplementary material for: Rapid prototyping of PMMA-based microfluidic spheroid-on-a-chip models using micromilling and vapour-assisted thermal bonding
Source: Sci Rep. 2024 Feb 3;14:2831. doi: 10.1038/s41598-024-53266-y (PMC10838337; doi:10.1038/s41598-024-53266-y)
Supplement: Supplementary file 1 — Supplementary Figures. [file 41598_2024_53266_MOESM1_ESM.docx]

**Supplementary Material**

**Rapid prototyping of PMMA-based microfluidic spheroid-on-a-chip models using micromilling and vapour-assisted thermal bonding.**

Monieb A. M. Ahmed, Klaudia M. Jurczak, N. Scott Lynn Jr, Jean-Paul S. H. Mulder, Elisabeth M. J. Verpoorte, Anika Nagelkerke^*^.

^*^Corresponding author: Pharmaceutical Analysis, Groningen Research Institute of Pharmacy, University of Groningen, Groningen, The Netherlands. [a.p.nagelkerke@rug.nl](mailto:a.p.nagelkerke@rug.nl)

ORCID iD:

Monieb Ahmed: 0000-0002-0884-7500

Klaudia Jurczak: 0000-0003-2346-2964

Scott Lynn: 0000-0002-8649-9214

Jean-Paul Mulder: 0000-0002-8893-6190

Elisabeth Verpoorte: 0000-0002-6616-4134

Anika Nagelkerke: 0000-0002-7541-588X

**Supplementary Materials and methods**

*The effect of surface roughness on cellular orientation*

A channel, 20 mm x 10 mm x 1 mm (l x w x h), was micromilled in PMMA. The channel was treated with chloroform vapour for 30 seconds and was sterilised with UV at wavelength of 300 nm. MDA-MB-231 cells were seeded on chloroform treated and untreated micromilled channels at cellular density of 30,000 cells/cm^2^.

*Finite element simulations of oxygen and glucose transport*

We used Comsol (v6.1) to obtain solutions to both (a) the steady state Navier-Stokes equations and (b) the steady state convection-diffusion equation. Simulations were performed in segregated fashion, where a solution to (a) was used as an input to obtain a solution for (b). An example simulation domain is shown in **Supplementary Figure 6**, where all necessary variable inputs are given in **Supplementary** **Table 1**. The domain regarding (a) consisted of the entire simulation geometry minus the spheroid cell mass, within which we considered fluid flow to be zero. The domain regarding (b) consisted of the entire simulation geometry, where the cell spheroid had an additional reaction condition where oxygen was consumed at a rate *R* given by Michaelis-Menten kinetics as $R=\frac{V_{max}C}{(K_{m}+C)}$, where *C* is the concentration of dissolved oxygen. The consumption of glucose was assumed to occur at the same rate as oxygen, where we assume aerobic respiration, where such consumption rate is only dependent on the oxygen concentration: the inlet concentration of oxygen (225 µM) is more than two orders of magnitude lower than the inlet concentration of glucose (25 mM). We assumed the diffusivity of oxygen and glucose was similar in both fluid and spheroid domains. The inlet consisted of a normal velocity input according to the volumetric flow rate *Q_in_* with a concentration *C_o_*. All reported simulations took advantage of the natural symmetry of the domain along the axial direction. Simulations consisting of shorter and longer inlet/outlet regions yielded similar results, as did non-symmetric simulations that included the lower microchannel (used for cell seeding, where for nutrient delivery there was no flow).

All numerical solutions were obtained using a multigrid method with successive overrelaxation for the pre- and post-smoother, and the PARADISO method for the course solver. Solutions for (a) and (b) were converged within 4 iterations using a 0.001 relative tolerance criterium.

The spheroid domain was meshed with tetrahedral elements having size Ms2, with the remaining fluidic domain having a mesh size of Ms1. The results of a mesh convergence study (to verify that solutions are accurate) are seen in **Supplementary Figure 6**, where we used a cell mass diameter of *d_cell_* = 0.8 mm (similar results were obtained with *d_cell_* = 0.4 mm). We first chose a relatively small mesh size of Ms2 = 0.023 mm to mesh the cell mass and observed changes in Ms1 on the numerical solutions. We observed that quadratic elements led to convergent behaviour of the outlet flow rate (matching within 2% of *Q_in_*) at mesh sizes below Ms2 = 0.1 mm. Using that fluidic mesh we then varied Ms1, where convergent behaviour for the overall O_2_ uptake (calculated via integrating the normal diffusive flux along the cell mass boundary) is seen for mesh sizes of Ms1 = 0.016 mm for simulations having quadratic elements for both (a) and (b). Using this mesh typical solutions consisted of 400k elements with 1.5M degrees of freedom for (a) and 0.5-1.3M degrees of freedom for (b), where simulations required roughly 8-11 GB of memory.

**Supplementary Data and Figures**

**Supplementary Movie 1**. MDA-MB-231 spheroid formation inside the microfluidic device over a period of two days.


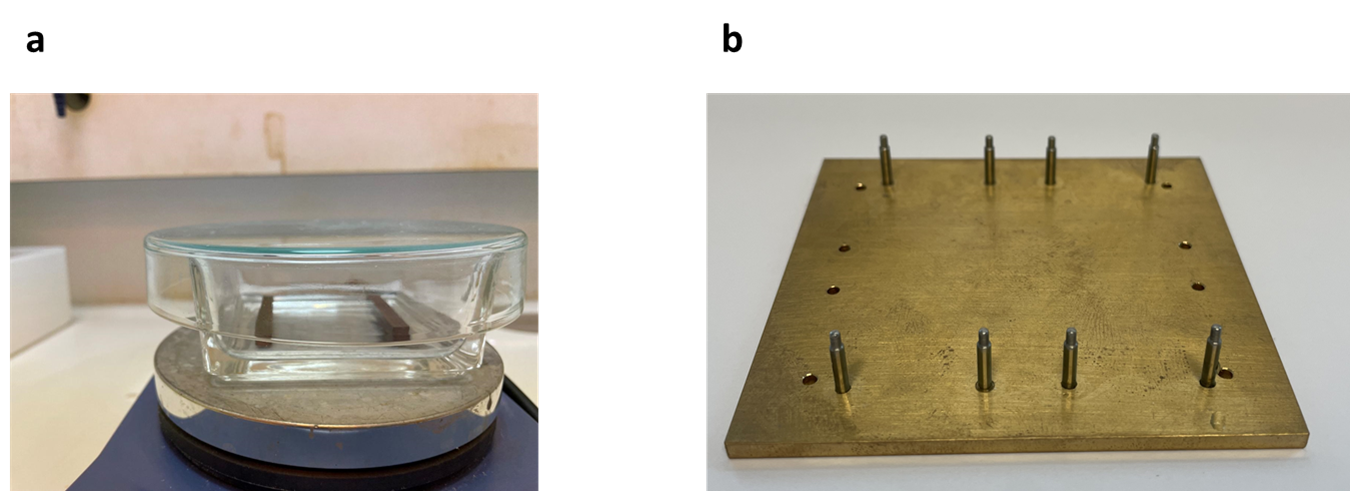


**Supplementary Figure 1.** Chloroform vapour chamber setup and alignment tool. (**a**) Micromilled PMMA substrates are positioned on metal pillars on top of the reservoir, with the micromilled channels facing the chloroform. (**b**) After the bonding procedure, PMMA based microfluidic chips are aligned together using a custom-built alignment tool.


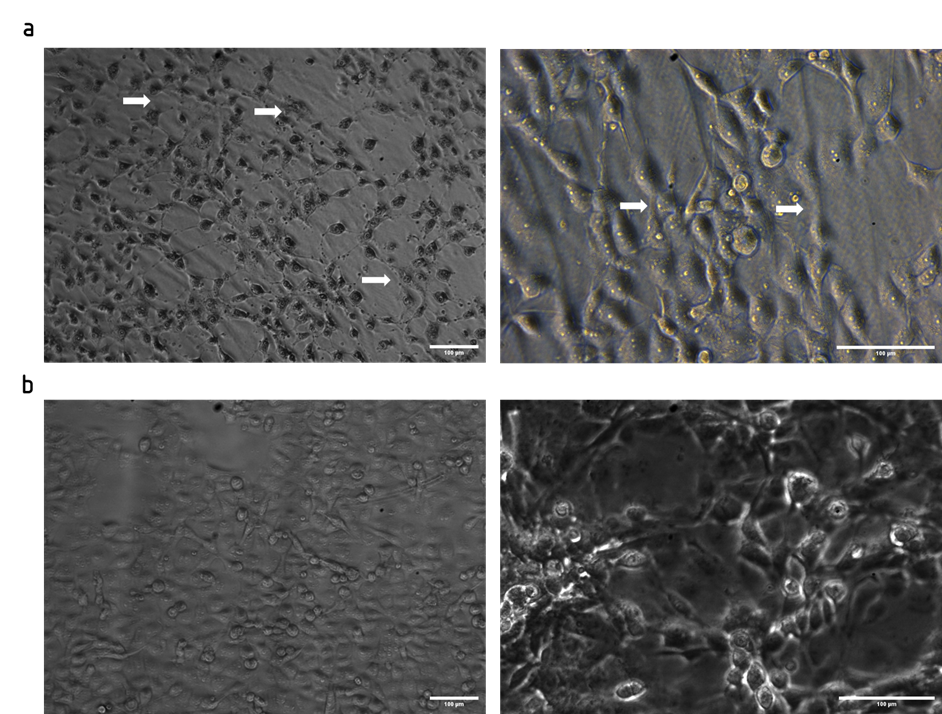


**Supplementary Figure 2.** MDA-MB-231 cells cultured on micromilled surfaces. Bright field images of cells cultured on micromilled surfaces before (**a**) and after (**b**) treatment with chloroform vapour. White arrows indicate cells oriented along milling tracks.


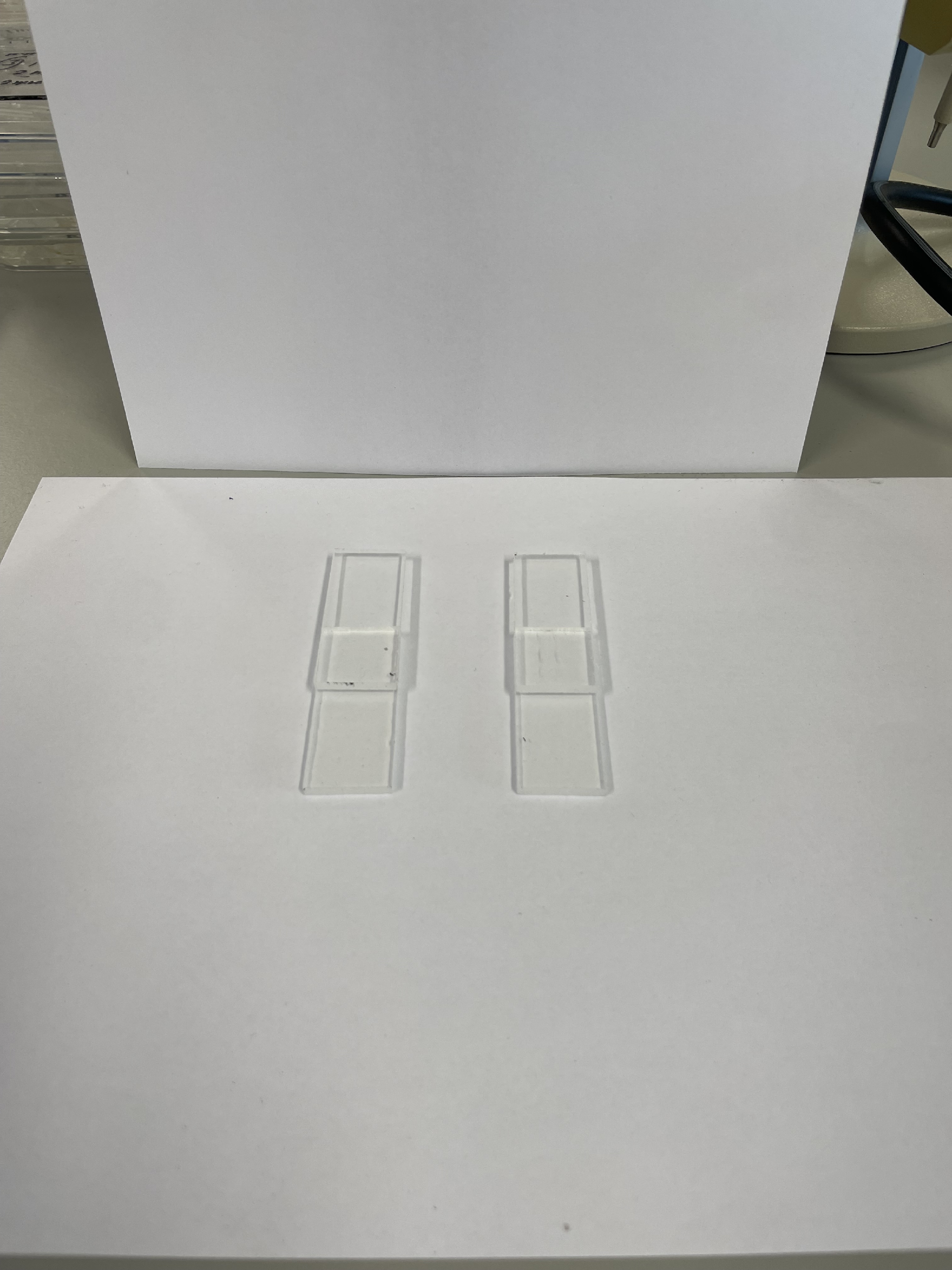


**Supplementary Figure 3.** Bonded PMMA substrates over an area of 4 cm^2^.


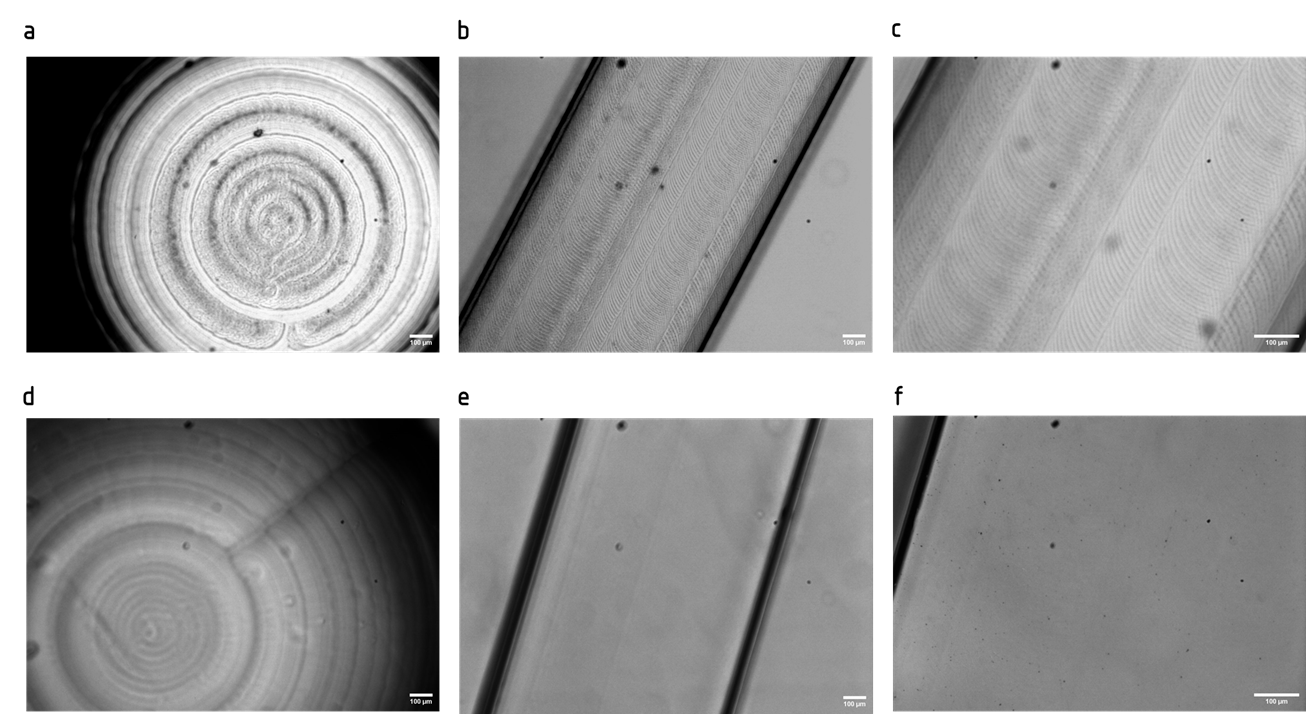


**Supplementary Figure 4.** Surface treatment and bonding of the spheroid-on-a-chip system. Representative bright field images of a micromilled well (**a**) and channel (**b** and **c**) before exposure to chloroform vapour. (**d**-**f**) show the effect of chloroform vapour on the well and channel.


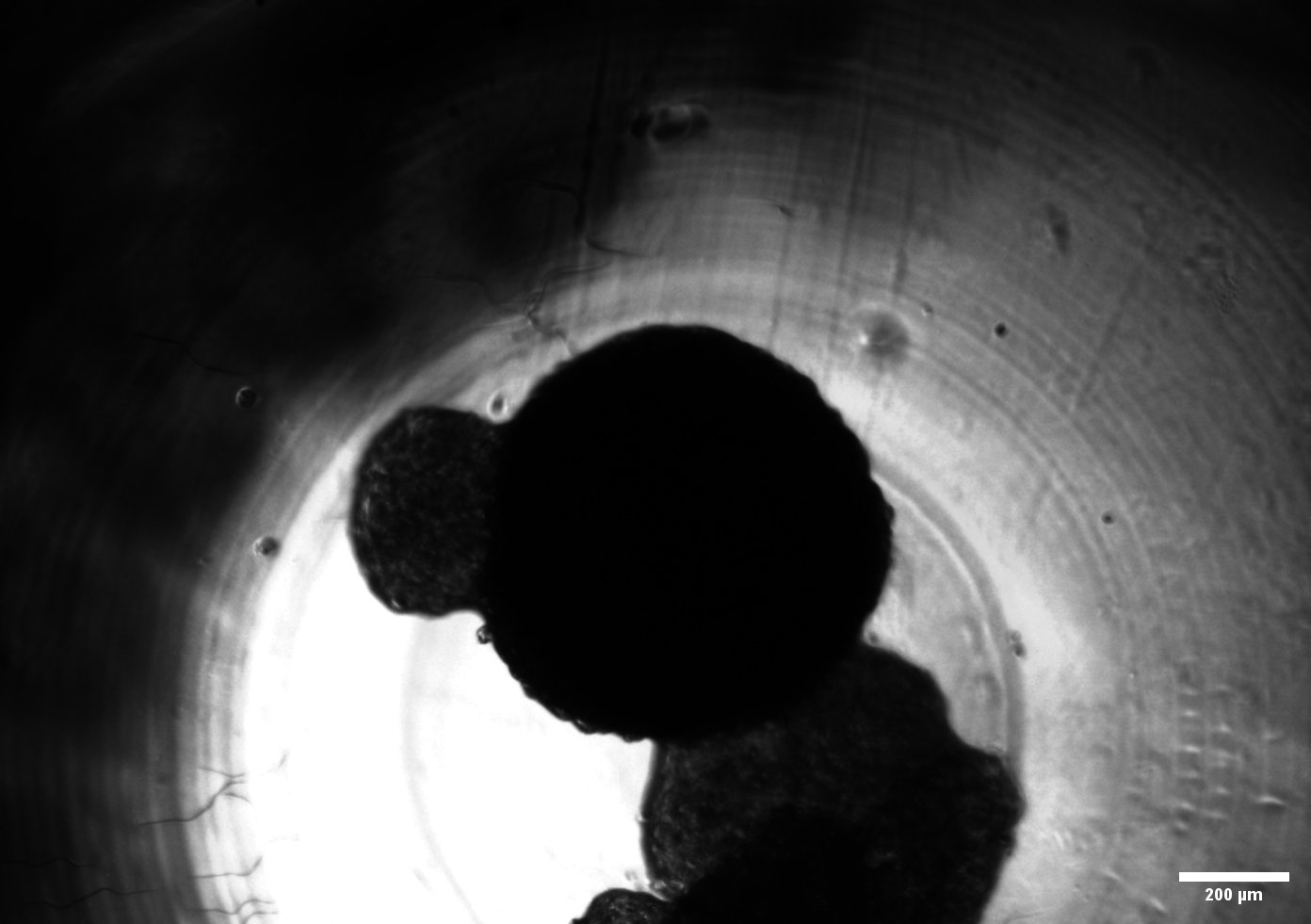


**Supplementary Figure 5.** Formation of MDA-MB-231 spheroid. After 7 days of culture, the formation of a MDA-MB-231 spheroid resulted in an irregular shape due to the presence of trapped cells in the channel. These cells formed smaller spheroids that interfered with the spheroid in the microwell.


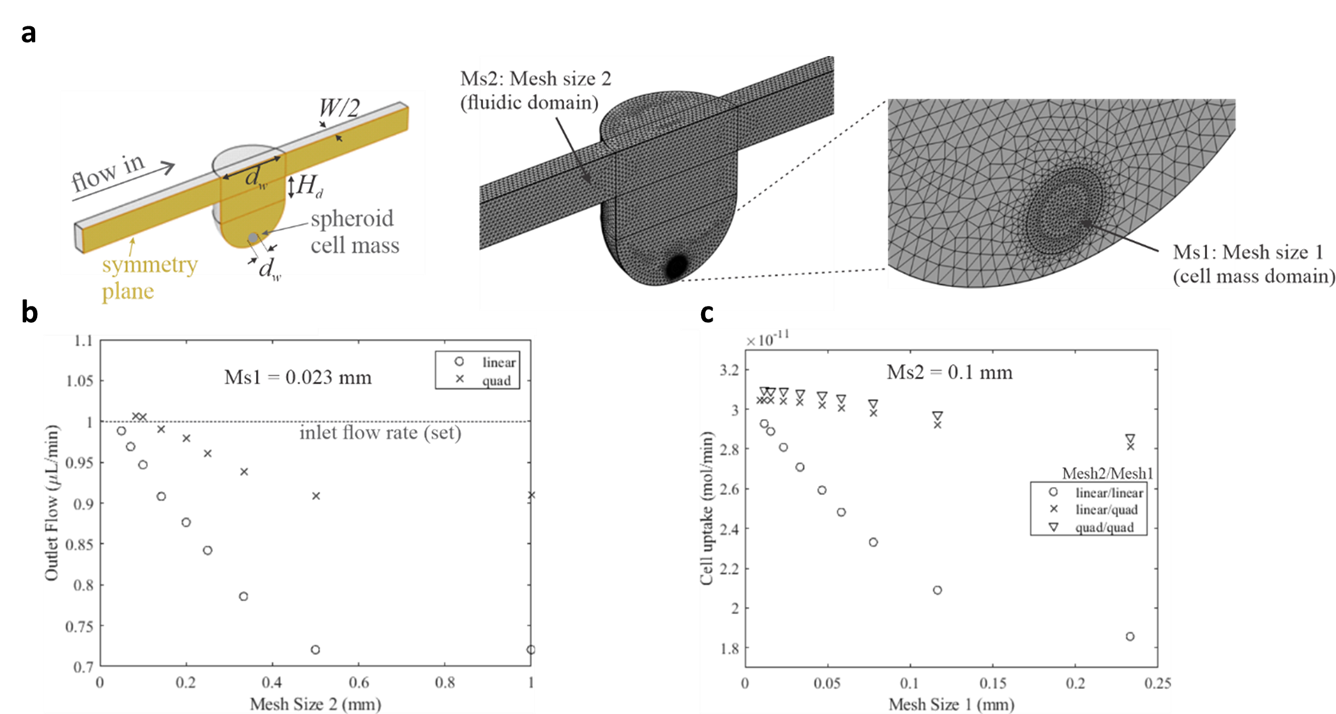


**Supplementary Figure 6.** Mesh convergence results. (**a**) The symmetric domain considered in the simulations for a cell mass diameter of *d_cell_* = 0.8 mm. The fluidic domain is discretized with a mesh having size Ms1 (constant Ms2 = 0.023 mm), where the domain representing the spheroid cell mass is discretized with a mesh having size Ms2. (**b**) Mesh test convergence test for the fluidic domain, where we selected the fluid flow at the outlet to assess the conservation of mass. It can be seen that the use of linear elements for the Navier-Stokes equations give poor convergence, whereas the use of quadratic elements (for the fluid velocity vectors) converges to values representing the inlet flow rate. (**c**) Mesh test convergence test for oxygen uptake by the cell mass, which was calculated by the normal diffusive flux of oxygen integrated across the outer cell boundary. We varied Ms1 (with optimal Ms2 = 0.1 mm) in simulations consisting of linear/linear elements (fluid flow/O_2_ transport), linear/quadratic elements, and quadratic/quadratic elements. Convergent behaviour can be seen for the quadratic/quadratic elements. Based on these results a fluidic domain discretization size of Ms1 = 0.1 mm (quadratic elements) and a spheroid mass discretization size of Ms2 = 0.016 mm (quadratic elements) was chosen.


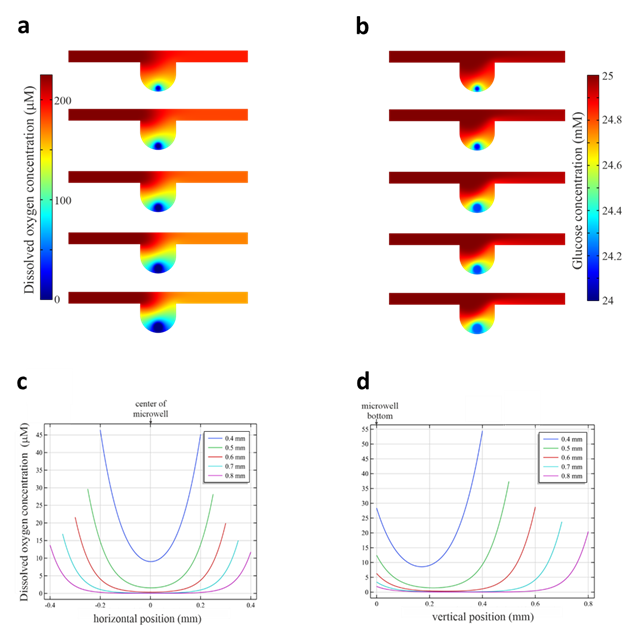


**Supplementary Figure 7**. Steady-state contours of (**a**) dissolved oxygen and (**b**) glucose. From these contour plots we plotted the concentration of dissolved oxygen within the spheroid in both the (**c**) horizontal and (**d**) vertical direction. These results follow those shown by Grimes *et al*. (whose results were used to calculate the reactive O_2_ uptake $V_{max}$ used herein), who showed that the O_2_ distribution for spheroid diameters below 0.466 mm will not reach zero, whereas spheroid diameters larger than 0.466 mm will have an inner anoxic region^4^. The major difference between these results and those by Grimes *et al*. is that they forced an equilibrium O_2_ concentration directly at the cell surface, where here the O_2_ concentration at the cell surface is dictated by both the well geometry as well as the inlet flow rate. Hence, the outer layers for each cell mass considered herein are exposed to an O_2_ concentration (along the vertical cell mid-plane) that is less than the equilibrium value of the inlet (255 μM).


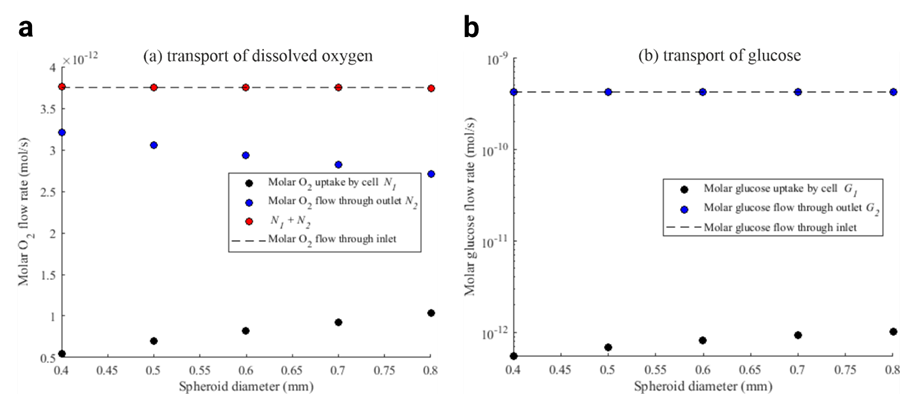


**Supplementary Figure 8**. (**a**) Molar O_2_ uptake by the cell (*N*_1_), through the outlet microchannel (*N*_2_), and the sum of the two (*N*_1_+*N*_2_) plotted as a function of the spheroid diameter. The molar flow rate increases slightly with increasing cell mass size; however, all rates remain well below the molar flow rate of oxygen through the input. For all simulations the overall molar loss of oxygen (i.e. the uptake by the spheroid plus the molar rate flowing through the outlet *N*_1_+*N*_2_) was within 0.1% of the inlet molar flow rate, which verifies the simulation method. (**b**) Molar glucose uptake by the spheroid (*G*_1_) and through the outlet microchannel (*G*_2_) plotted as a function of the spheroid diameter. The rate of glucose uptake by the spheroid is less than 2 orders of magnitude less than that provided by the inlet microchannel.

**Supplementary Table 1**. Variables and equations used in the simulations

| **Description** | **Parameter** | **Value** |
| --- | --- | --- |
| Inlet flow rate | *Q_in_* | 1 µL/min |
| Inlet channel width | *W* | 1 mm |
| Inlet channel height | *H* | 1 mm |
| Well diameter | *d_w_* | 3 mm |
| Displacement height | *H_d_* | 1 mm |
| Cell mass diameter | *d_cell_* | variable |
| Inlet O_2_ concentration | *C_o_* | 225 µM ^1^ |
| Growth media viscosity | *µ* | 0.930 mPa∙s ^2^ |
| Growth media density | *ρ* | 1.009 g/cm^3 2^ |
| Temperature | *T* | 310.15 K |
| O_2_ diffusivity | $D$ | 2.41×10^-9^ m^2^/s ^3^ |
| Reactive uptake | *V_max_* | 1.81 mol/m^3^/min ^4^ |
| Michaelis-Menten constant | *K_m_* | *C_o_*/10 |
| Glucose diffusivity | *D_g_* | 5×10^-10^ m^2^/s |
| Inlet Glucose concentration | *C_g_* | 25 mM |
|  |  |  |
|  |  |  |
|  |  |  |

**References**

1 Brennan, M. D., Rexius-Hall, M. L., Elgass, L. J. & Eddington, D. T. Oxygen control with microfluidics. *Lab Chip* **14**, 4305-4318, doi: 10.1039/c4lc00853g (2014).

2 Poon, C. Measuring the density and viscosity of culture media for optimized computational fluid dynamics analysis of in vitro devices. *J Mech Behav Biomed Mater* **126**, 105024, doi: 10.1016/j.jmbbm.2021.105024 (2022).

3 Wilke, C. R. & Chang, P. Correlation of diffusion coefficients in dilute solutions. *AIChE Journal* **1**, 264-270, doi: 10.1002/aic.690010222 (1955).

4 Grimes, D. R., Kelly, C., Bloch, K. & Partridge, M. A method for estimating the oxygen consumption rate in multicellular tumour spheroids. *J R Soc Interface* **11**, 20131124, doi: 10.1098/rsif.2013.1124 (2014).
